# Erythema multiforme major with ocular involvement following COVID-19 infection

**DOI:** 10.1093/omcr/omab120

**Published:** 2021-12-28

**Authors:** Carla L Maden, Laura Ah-Kye, Yasmin Alfallouji, Elizabeth Kulakov, Peter Ellery, Esther Papamichael

**Affiliations:** 1 FY2, Imperial College Healthcare NHS Trust, London, UK; 2 Ophthalmology Department, Royal Free London NHS Foundation Trust, London, UK; 3 Dermatology Department, University College London Hospitals NHS Foundation Trust, London, UK; 4 Histopathology Department, University College London Hospitals NHS Foundation Trust, London, UK

## Abstract

We report a case of membranous conjunctivitis and erythema multiforme major (EMM) after a coronavirus disease 2019 (COVID-19) diagnosis. A previously well 18-year-old man presented with increasingly erythematous eyes and oral and genital ulceration 2 weeks after confirmation of COVID-19 infection. Clinical examination showed sloughy membranous conjunctivitis with normal visual acuity. He was reviewed by dermatology and diagnosed with EMM secondary to severe acute respiratory syndrome coronavirus 2 infection. The symptoms resolved with oral and topical steroids, lubricants and chloramphenicol eye drops. Erythema multiforme has been reported in association with COVID-19, although the major form is rare. Ophthalmologists should consider current or previous COVID-19 infection in patients presenting with conjunctivitis or pseudomembrane formation. Prompt initiation of steroids aids resolution.

## INTRODUCTION

Our understanding of the presentation of coronavirus disease 2019 (COVID-19) has advanced significantly since its identification in December 2019. Classical symptoms include fever, cough and shortness of breath but other clinical manifestations have since been described including associated dermatological and ocular manifestations. Common cutaneous signs include urticarial, erythematous and papulovesicular rashes [1], whereas dry eye and conjunctivitis are the most frequently reported ophthalmic signs [2].

Erythema multiforme (EM) in association with COVID-19 has been documented in several case reports, but most present with a minor form of the disease and ocular involvement has been rare. We report a case of bilateral bulbar membranous conjunctivitis in a patient with severe acute respiratory syndrome coronavirus 2 (SARS-CoV-2) related erythema multiforme major (EMM).

## CASE REPORT

**Figure 1:**
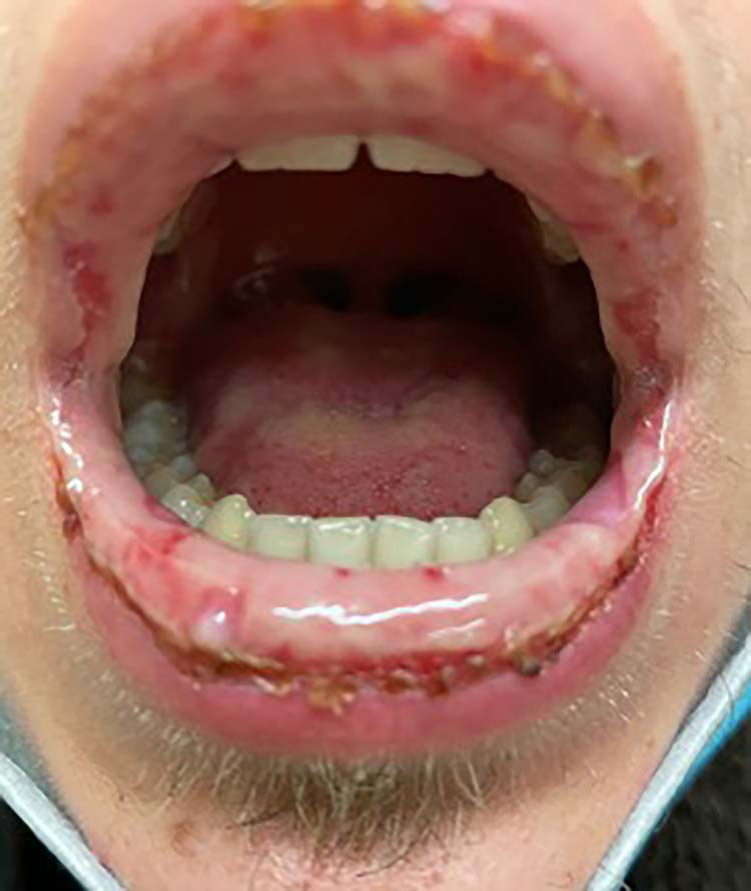
Mouth ulcers.

A previously fit and well 18-year-old man presented to the eye casualty with 7 days of increasingly red eyes, no discharge, a feeling of heaviness around the eyes, and oral and penile ulcers. He had been diagnosed with COVID-19 3 weeks earlier and had symptoms of fatigue, anosmia, headache, shortness of breath and sore throat. The eye symptoms and ulcers came on after resolution of his COVID-19 symptoms. On Day 1 of his eye symptoms, he had a remote consultation with a tertiary eye casualty and was given lubricating eye drops for presumed viral conjunctivitis. A day after this, he was prescribed oral penicillin V for a sore throat and developed the penile and mouth ulcers as soon as he took the antibiotics (Fig. 1). His general practitioner (GP) referred him to dermatology and rheumatology for investigation.

**Figure 2:**
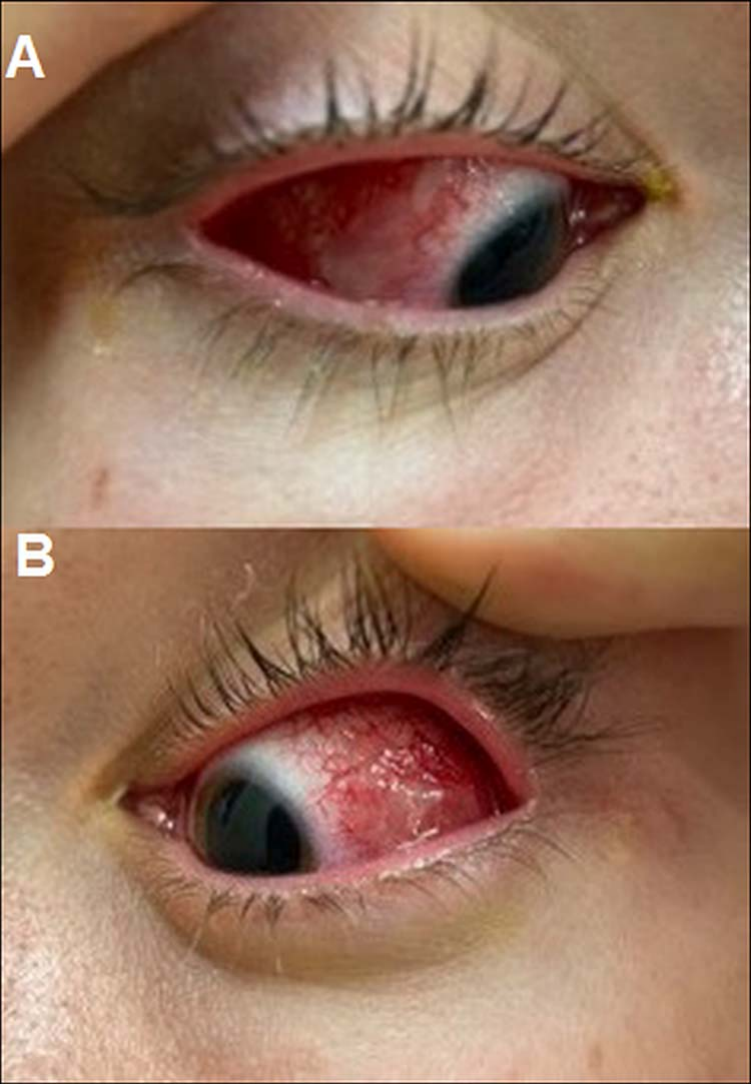
(**a**) Right eye membranous conjunctivitis. (**b**) Left eye membranous conjunctivitis.

**Figure 3:**
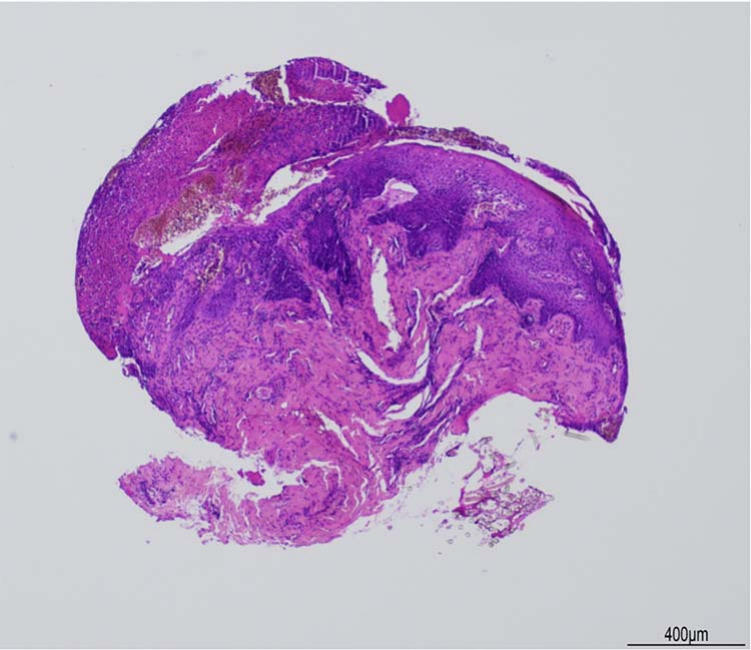
Haematoxylin & eosin (H&E) stain, **×**4 objective. Histology shows a small, partly-crushed biopsy of ulcerated squamous-lined mucosa with parakeratosis and irregular squamous hyperplasia.

**Figure 4:**
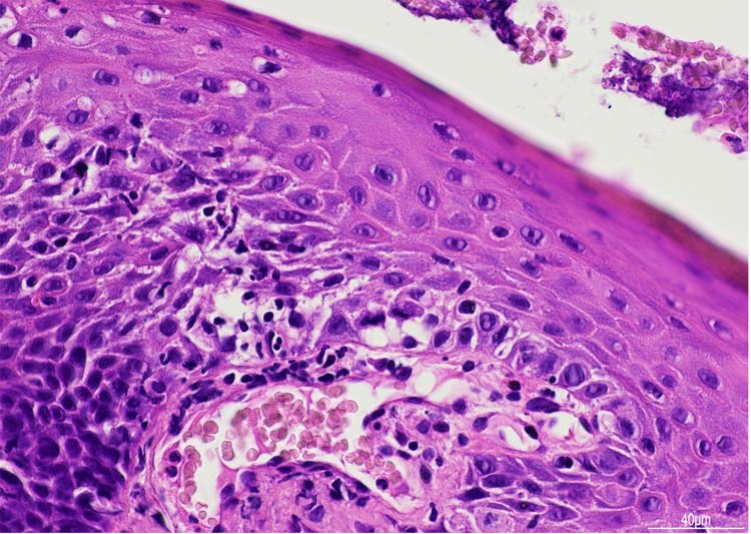
H&E stain, **×**40 objective. There is mild lymphocyte exocytosis and associated basal vacuolar degeneration, in keeping with focal interface mucositis.

He was referred for review in eye casualty from the dermatology clinic, and presented 7 days after the red eyes and 6 days after the oral and genital ulceration had begun. On examination visual acuity was 6/6 in the right and 6/9 in the left. There was diffuse injection of the bulbar conjunctiva with bilateral conjunctival pseudomembranes, but no tarsal conjunctival epithelial defect on eversion of the lid (Fig. 2). The corneas were clear with no epithelial defects. He was started on copious lubrication, 2 hourly preservative-free dexamethasone 0.1% drops, and preservative-free chloramphenicol drops 4 times a day as prophylaxis. Two days later, his symptomatology improved, and the bulbar conjunctival injection and pseudomembranes improved significantly. The patient was reviewed again by dermatology and diagnosed with SARS-CoV-2 associated EMM following the results of a 3-mm punch biopsy of the lower lip, which showed ulcerated skin with focal interface mucositis (Figs 3 and 4). He was started on prednisolone 30 mg orally for 1 week. He was advised to continue the chloramphenicol and taper down the dexamethasone drops. Two weeks later he made a full recovery. There were no ophthalmic signs and no remaining ulcers. He was advised to stop the chloramphenicol and taper off the dexamethasone drops gradually.

## DISCUSSION

EM is a hypersensitivity reaction most commonly associated with herpes simplex and mycoplasma pneumoniae infections but can also be drug-induced [3]. The major form is characterized by prodromal symptoms, blistering and ulceration of mucous membranes. Ocular complications can be sight-threatening and include keratitis, uveitis and conjunctival scarring [4]. Here, we present a case of EMM associated with recent SARS-CoV-2 infection. The ocular symptoms occurred prior to the start of a course of penicillin V, making this an unlikely trigger, although it is possible it may have contributed to the symptom severity.

EM is an uncommon cutaneous manifestation of COVID-19; COVID-19 related EM has predominantly been reported in children [5–7]. We found only two case reports of COVID-19 related EM with ocular involvement, which were reported as a very mild conjunctival injection [7, 8]. To our knowledge this is the first case reporting a severe membranous conjunctivitis. Management is aimed at reducing ocular inflammation with topical steroids, lubricating the ocular surface and topical antibiotics if the ocular surface epithelium is compromised. This case highlights the consideration of COVID-19 associated EM as a diagnosis in a patient presenting with membranous conjunctivitis and mucosal ulceration.
